# Investigation of changes in the profile of fatty acids and growth traits in response to waterlogging/flooding stress

**DOI:** 10.1371/journal.pone.0355293

**Published:** 2026-08-28

**Authors:** Muhammad Zubair Gul, Mudassar Nawaz Khan, Tariq Mahmood, Sumaira Salahuddin Lodhi, Muhammad Tayyab, Nisar Ahmad, Manan Khan, Iftikhar Ahmed, Muhammad Akram

**Affiliations:** 1 Institute of Biotechnology and Genetic Engineering, The University of Agriculture, Peshawar, Pakistan; 2 Department of Biotechnology and Genetic Engineering, Hazara University, Mansehra, Pakistan; 3 Department of Agriculture, Hazara University, Mansehra, Pakistan; 4 Department of Biochemistry, Hazara University, Mansehra, Pakistan; 5 Land Resources Research Institute, National Agricultural Research Centre, Islamabad, Pakistan; 6 Medicinal Botanic Centre, Pakistan Council of Scientific and Industrial Research Labs Complex, Peshawar, Pakistan; Yeungnam University, KOREA, REPUBLIC OF

## Abstract

Abrupt climatic changes around the globe have brought serious challenges for agriculturally important crops due to increase in environmental temperature and frequent flooding/waterlogging events. Flooding acts as abiotic stress on plants by challenging the both growth and yield. Wheat is exposed to abiotic stresses such as flooding but its response is less studied. In the current study, Atta Habib, a local wheat variety, was analyzed for its response to flooding stress and post-flooding recovery. The lengths and weights of the roots and shoots, as well as the relative water content, ascorbate peroxidase activity, and fatty acid contents, were analyzed. Twenty-one days-old wheat seedlings were flooded for 7 days and then allowed to recover for 7 days following flooding removal. The lengths of the flooded shoots were retarded slightly to 15.62 cm as against 16.49 cm in age-matched control-1 seedlings (6% decrease). The weight in flooded shoots was measured as 199.37 mg, as against 212.27 mg in control-1 seedlings (6% decrease). Flooding retarded the root length to 12.99 cm that was 14.86 cm in control plants (14% decrease). Root weight was significantly reduced to 78.4 mg in flooded plants which was 138.33 mg in control plants (76% decrease). During post-flooding recovery stage, shoot length & weight were recovered to 4% and 7%, respectively as compared to flooded plants. Similarly, root length & weight showed 14% and 32% recovery, respectively during the post-flooding stage. Relative water content increased significantly to 92.38% in flooded shoots in comparison to control-1 seedlings. Ascorbate peroxidase activity was decreased by 11% in the post-flooding recovery stage. Linolenic acid content was 51.01% in control-1 plants, increased slightly to 56.82% in flooded wheat, and was reduced to 43.21% during recovery period. Palmitic acid was quantified as 34.54% in control-1, increased slightly to 34.95% in flooded seedlings, and increased noticeably to 37.76% during recovery. Linoleic acid (7.41%) and stearic acid (5.30%) were increased only during recovery. Oleic acid increased significantly in flooded (2.26%) and recovering seedlings (11.51%) in comparison to 0.95% and 2.60% in control-1 and control-2 seedlings, respectively. Alterations in growth parameters, ascorbate peroxidase activity, and fatty acids content reveals the adjustments in response to flooding stress, as a way to cope with the stress as shown by recovery trend; revealing that plants adjusted the analytes in post-flooding recovery stage. The adjustments in biochemicals under the flooding stress and during recovery point out their crucial role in flooding stress response. It is also proposed that future studies can be expanded with diverse germplasm and flooding stress durations to reveal more information.

## 1. Introduction

The increasing global population has led to a heightened focus on wheat (*Triticum aestivum* L.) on a global scale [1]. Wheat, which is composed of 58.2% carbohydrate and sufficient quantities of body lipids and sugars, is an excellent source of energy. In 2024−25, 799.30 million metric tonnes of wheat was produced worldwide, with the United States, Australia, Canada, China, India, and Russia being the leading producers [2]. Wheat is a staple grain in Pakistan and has a share of 1.9% in GDP and 8.7% in value addition of agricultural products. According to the Pakistan Economic Survey 2024−25, country produced 28.98 million tonnes of wheat [3].

Wheat production faces several biotic and abiotic factors that decrease its final yield. Drought, salinity, heat, cold, UV light and flooding/submergence/waterlogging all act as abiotic stresses on plants. Among these, flooding is one of the main abiotic stresses for agricultural crops in the current era of abrupt climatic changes. It is characterised by the presence of water in excess of its minimum requirements [4]. Floods have affected about 16% of agricultural land, resulting in sizable economic losses [5]. Flooding affects 10–15 million hectares of wheat crop, resulting in 20–50% yield losses [6]. Floods are second largest disaster to agriculture, causing damages to 27% of cultivated land annually [7]. In 2010, floods resulted in 4.45 billion dollars losses for wheat, cotton, and rice harvests in Pakistan [8]. Recent floods in 2022 in Pakistan have caused havoc to the whole country, and initial damages estimate 60 billion dollars in losses to the economy. Crops worth billions of dollars have been swept away. Flooding is a multi-pathway involving stress that leads to metabolic, chemical and nutritional re-arrangements in plant [9]. Flooding stress leads to low-oxygen conditions such as hypoxia and anoxia [10]. Flooding induce an oxidative damage to the plants that leads to reactive oxygen species (ROS) accumulation [11]. Wheat is a vulnerable to flooding stress as documented in many studies [12]. Flooding/waterlogging on wheat adversely affects plant’s photosynthesis, respiration, transpiration, antioxidative system, enhances senescence, and thus decreases yield [13–16]. Photosynthesis which is directly related to yield, was reported a 31% decrease in flooded wheat [17]. Flooding-led ROS accumulation in wheat causes lipid peroxidation that damage cell membrane, nucleic acids and proteins which may lead to cell death [6]. Flooding alters concentrations of sugars and organic acids and so affects the nutrient quality [18]. Decreased potential of recovery was recorded in wheat following flood removal [16]. Short and long flooding durations have versatile effects on wheat growth ranging from stem weakening to yield losses [19].

Plants respond to flooding stress on multi-fronts. The response of plants to flooding may be either through escape strategy or quiescence strategy [5], but these strategies are differentially prioritized across species [20]. Wheat response to flooding depends on flooding duration and growth stage [19]. During floods, plants increase glycolysis and the activity of fermentation-linked enzymes, including pyruvate decarboxylase and alcohol dehydrogenase. In contrast, the levels of ROS scavengers, such as peroxidase and superoxide dismutase, decrease [21]. It has been shown that ascorbate peroxidase (APX) can reduce the oxidative damage that occurs as a result of flooding [22]. Peroxidase (POD) activity in flooded soybeans increased during the post-flooding recovery period [23]. Higher levels of the pyruvate decarboxylase gene in transgenic Arabidopsis led to production of more ATP and NAD+, that made the plants more resistant to waterlogging stress [24]. Flooding stress increased the hydrogen peroxide and malondialdehyde contents in leaves and reduced their chlorophyll content, photosynthetic efficiency, and shoot dry weight. In addition, flooding increased the activities of catalase and POD [25]. Genes that were differentially expressed under flooding stress, included those related to phenyl-propanoid biosynthesis, and plant hormone signal transduction pathways [26]. Moreover, ethylene response factors vii were reported to be involved in flooding-induced hypoxia sensing and signaling [25].

Plants respond to abiotic stresses by using lipid signalling mechanisms [27]. In addition to metabolic changes, preservation of root architecture, and cytoskeleton organisation, lipid messenger molecules play a crucial role in facilitating adaptive responses to osmotic circumstances [27,28]. Plants subjected to oxidative difficulties, exhibited elevated amounts of palmitic acid, phosphatidylinositol, and oxylipin [29,30]. Hypoxia arising due to flooding stress negatively impacts plant cellular homeostasis by decreasing oxidative phosphorylation in mitochondria, leading to a reduction in cellular energy charge [31]. Acyl-CoA activation needs ATP, which changes how newly made fatty acids get from the plastid to the cytosol [32]. Soybean utilises energy-efficient processes and induces biochemical and structural changes in the cell wall to efficiently combat flooding stress [33].

Metabolites are the small molecules that are the part of gene expression, protein correlation, and many regulatory processes. Metabolites have a stronger correlation with the phenotype compared to messenger RNA transcripts or proteins alone [34]. Metabolomics has emerged as useful tool to link metabolic pathways to plant responses to biotic and abiotic stresses. Integrating metabolomics with modern tools, has paid the way to reveal the association b/w genetics and phenotypes in crops [35,36]. Under challenging circumstances, plants accumulate primary metabolites such as osmolytes, osmo-protectants, and secondary metabolites (protective metabolites) to enhance their ability to withstand stress [37]. Amino acids levels were reported to accumulate in wheat under flooding stress; especially those involved in gamma amino butyric acid shunt and anaerobic/aerobic metabolism [38]. Metabolomics integrated with genomics revealed that Shikimate pathway is involved in plant responses to stresses, through production of aromatic amino acids, phenolics, flavonoids, lignin and alkaloids [39]. Metabolites such as small acids, alcohols, amino acids, fatty acids, hydroxyl acids, sugars, catechol-amines, sterols, toxins, and medicines are detected and measured using gas chromatography-mass spectrometry [40]. Gas chromatography-mass spectrometry (GC-MS), an exceptional analytical method that enables precise separation and resolution, is used in targeted and untargeted metabolomics. The GC-MS technique provides analysis of a variety of metabolites in a relatively proportionate manner [41].

Fatty acids are the metabolites that are structural parts of the plasma membrane as well as other cellular membranes in all living cells. Fatty acids play an important role in signaling events that regulate metabolic processes. Plant cell membranes have structural glycerol-lipids with 16-carbon and 18-carbon fatty acids [42]. Fatty acids are important parts of suberin and cutin waxes, as well as the membranes of cells and organelles. They protect against stress from both living and nonliving things [43]. Fatty acids which are key building blocks of many lipids, play direct and indirect essential roles in plant physiology and defence. These include linoleic acid, and α-linolenic acid. Also the compositions of galactolipids, phospholipids, and sphingolipids show responses to hypoxia-related stresses [44,45]. Anoxia resulting from submergence, led to massive lipid degradation in membranes of *Arabidopsis thaliana* [46]. Lipidomic profiling of *Argania spinose* L. (Skeels) ecotypes under drought stress revealed significant changes in 21 fatty acid metabolites, of which many were up-accumulated. Alpha linolenic acid was accumulated to the maximum [47]. Lipidomic analysis on heat-stressed wheat reported significant rise in digalactosyl diacylglycerol and phosphatidylinositol in the heat-tolerant cultivar. However, the significant decrease was noted in sulfoquinovosyl diacylglycerol, monogalactosyl monoacylglycerol, and phosphatidic acid [48]. Fatty acid content and composition significantly changed in ramie leaves under submergence stress; specifically, fatty acid content accumulated with increasing duration of submergence [49]. Stress-induced hypoxia increased the triacylglycerol and unsaturation in tomato roots [50]. The above mentioned studies clearly reveal the involvement of lipids/fatty acids in various abiotic stress responses.

The studies regarding wheat responses to flooding stress are a few; and those analysing fatty acid roles in flooding stress in wheat are almost non-existing. There is a dire need to explore flooding response mechanism in wheat so as to understand and develop flooding-tolerant cultivars in these challenging climates. Owing to multiple roles of lipids/fatty acids in normal physiological processes, signaling and stress responses, the need to explore their possible involvement is flooding stress responses will be a novel and interesting avenue. On the same front, rare studies exist on the post-flooding recovery potential of plants in general and wheat in particular. With these aims, the current study investigated wheat rresponses as well as its possible efforts to recover after flooding removal. The analyses were conducted at morphological, enzymatic, and GC-MS-based fatty acids metabolite levels.

## 2. Materials and methods

### 2.1. Plant germination and waterlogging/flooding stress treatment

**Fig 1:**
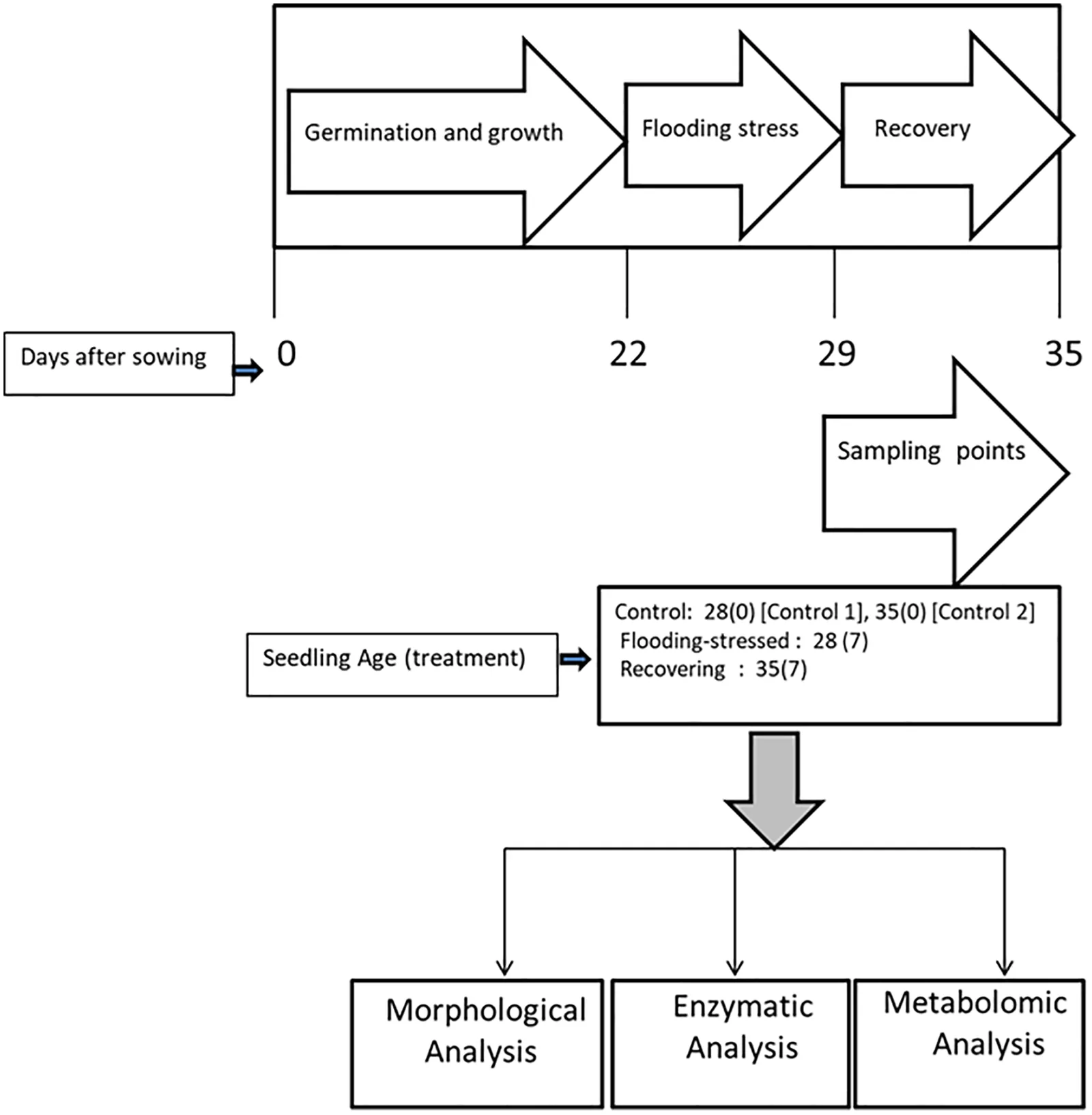
Experimental design of the study. Wheat seeds were sown and cultivated until the 21^st^ day. The seedlings were subjected to flooding stress for 7 days, followed by a 7-day recovery period. Control plants were sampled in addition to plants experiencing flooding stress and recovery. The 28(0) and 35(0) are control 1 and control 2 plants; whereas 28(7) and 35(7) are the flooding-stressed and recovering plants after 7 days flooding, respectively.

Seeds of the wheat variety; Atta Habib, were planted in small pots in a greenhouse installed at the Institute of Biotechnology and Genetic Engineering, University of Agriculture Peshawar, Pakistan in November 2022. Each pot was sown with 7–8 seeds, and there were 16 pots in each replication. For each sample type, 4 pots were used to grow and collect samples. Three replications of the samples were used for morphological, biochemical, and metabolite studies. After 21 days of planting (0–21; 22 days old), flooding stress was imposed for 7 days, while a few plants of the same age were kept as control. Pots with seedlings were placed in huge buckets holding 6 liters of water to induce flooding stress. Water was drained after 7 days, and certain pots with plants that had been stressed by flooding, were retained for a 7-day recovery period in a normal environment. The samples taken following flooding stress were a control sample (Control-1, after 28 days of sowing) and a flooded sample 28(7). Two types of samples collected after a 7-day recovery period were a recovery sample 35(7), and a control sample (Control-2, after 35 days of sowing), as explained in Fig 1.

### 2.2. Phenotypic and morphological characterization

For observing phenotypic changes in the collected samples at each time point (control-1, flooded, control-2, recovery), photographs were taken with the camera. The lengths and weights of the shoot and root of control, flooded, and post-flooding recovering plants were measured through a scale and weighing balance. The entire plant, including its roots, was carefully removed to prevent any damage to the roots. The soil was removed from the roots by rinsing them with clean tap water.

### 2.3. Relative water content

The relative water content (RWC) of the flooded and control plants was determined following formula [51].


$$\begin{array}{c} \text{RWC}\%=\frac{\left(FW-DW\right)}{\left(SW-DW\right)}\times \text{1}\text{0}\text{0} \end{array}$$

Where FW represents fresh weight, DW represents dry weight, and SW represents the saturated weight of the wheat shoot. The shoot’s saturated weight was determined by placing it in a water bath under light at 22˚C until it acquired a consistent weight (after 4 hours) and became fully turgid. The shoot’s dry weight was determined by placing the turgid shoot in an oven at 80˚C for 18 hours.

### 2.4. Ascorbate peroxidase (APX) assay

A 200 mg of the obtained sample was thoroughly mixed in 2.5 mL of 25 mM potassium phosphate buffer (pH 7.8) with 2% polyvinyl-pyrrolidone, 0.4 mM EDTA, and 1 mM ascorbic acid. The solution underwent centrifugation at 15,000g for 20 minutes at 4°C, and the resulting clear supernatant was collected to measure APX activity. The protein content was determined using the Bradford test with bovine serum albumin as a reference [52]. A reaction mix with 25 mM potassium phosphate buffer (pH 7.0), 0.25 mM ascorbic acid, 0.4 mM EDTA, and 0.1 mM H_2_O_2_ was used to measure the activity of APX. The APX activity was measured by monitoring the decrease in absorbance at 290 nm with a UV/Vis Spectrophotometer [53].

### 2.5. Fatty acids’ metabolomics through gas chromatograph-mass spectrometer

The fatty acid composition was analyzed using a gas chromatograph-mass spectrometer (GC-MS) QP2010 Plus Shimadzu (Japan). The methodology outlined by [54] was utilized for extracting and preparing both standard and sample materials. A 100 mg of the plant sample was extracted using 2 mL of petroleum spirit and ground with mortar and pestle for fatty acids extraction. At ambient temperature, the samples were centrifuged at 5000 rpm for 5 minutes. After centrifugation, 1 mL of clear supernatant was transferred to another tube for trans-methylation. Next, 0.5 mL of a sodium methylate solution (10g of CH_3_ONa in 500 mL of methanol) was added and incubated at room temperature for 30 minutes to complete the trans-methylation reaction. A 0.5 mL of 1M NaCl solution was then as the final step of sample preparation, resulting in the formation of two distinct layers after 5 minutes. The top layer was utilized for additional investigation of fatty acids using GC-MS.

The following conditions of GC-MS were maintained: column temperature: 50°C held for 1 min, increased to 150°C at the rate of 5°C/min, then increased to 175°C at the rate of 2°C/min and held for 5 min, finally increased to 220°C at the rate of 10°C/min and held for 5 min. The injector temperature was set at 250°C, while the ion source and interface temperatures were set at 240°C. MS was run in scan mode with an event time of 0.50 sec. The m/z ranged from 85 to 380. Peaks responding to fatty acid methyl esters (FAMES) were identified by searching against NIST mass spectral library.

### 2.6. Statistical analysis

For statistical analysis of the results, One-way ANOVA was used followed by Tukey’s HSD post-hoc test.

## 3. Results

### 3.1. Effect of flooding stress on phenotype of wheat

**Fig 2:**
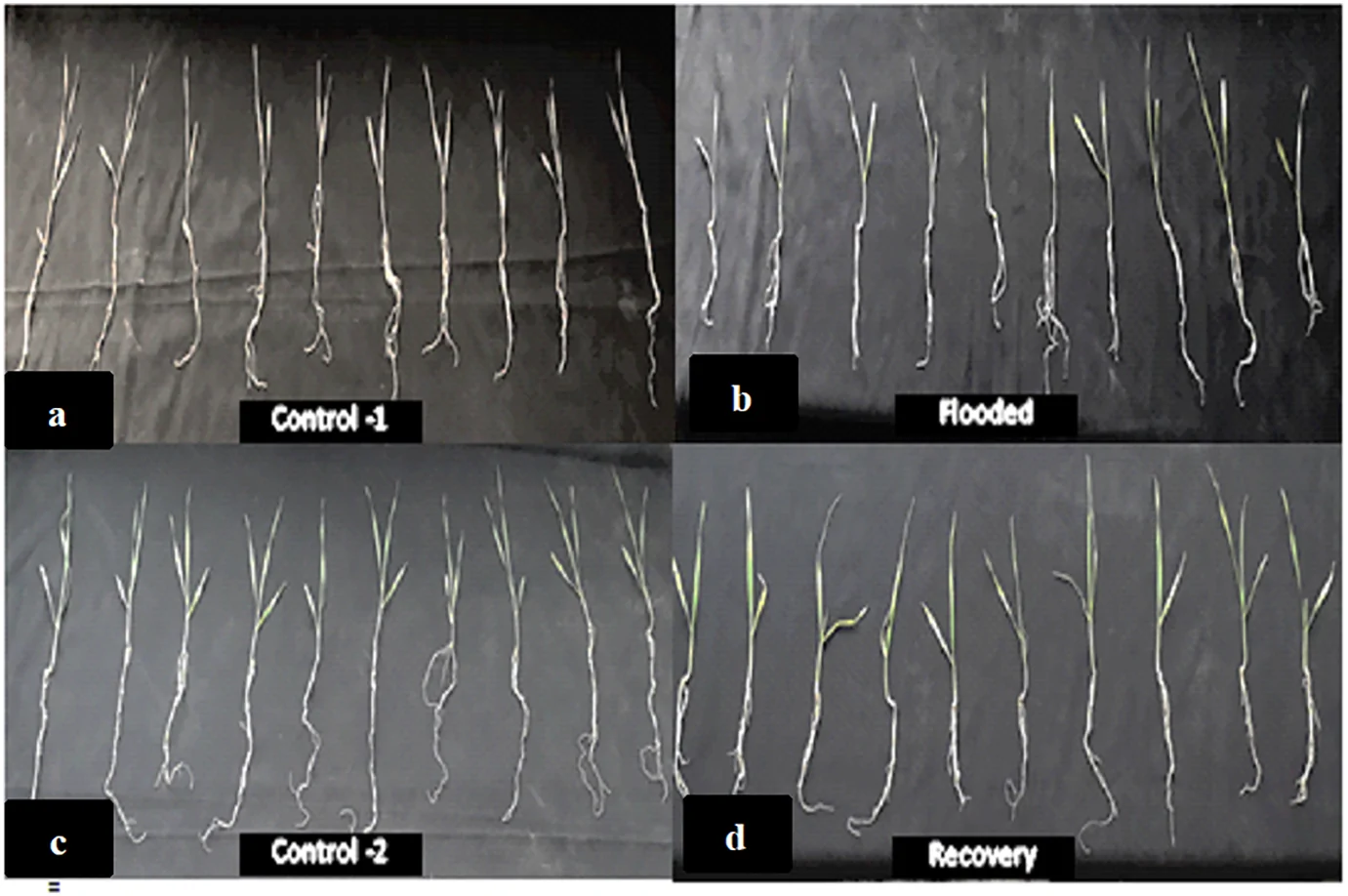
Changes in shoot’s pigmentation under flooding stress. a: control 1 [28(0)], b: flooding-stressed [28(7)], c: control 2 [35(0)], d: recovering [35(7)].

The wheat leaves of the control plants exhibited a green color. The shoots became pale yellow due to flooding stress. Following the removal of flooding stress (recovery phase), the shoot color turned lighten green as revealed in Fig 2.

### 3.2. Morphological changes in shoot length and weight under flooding stress and recovery durations

**Fig 3:**
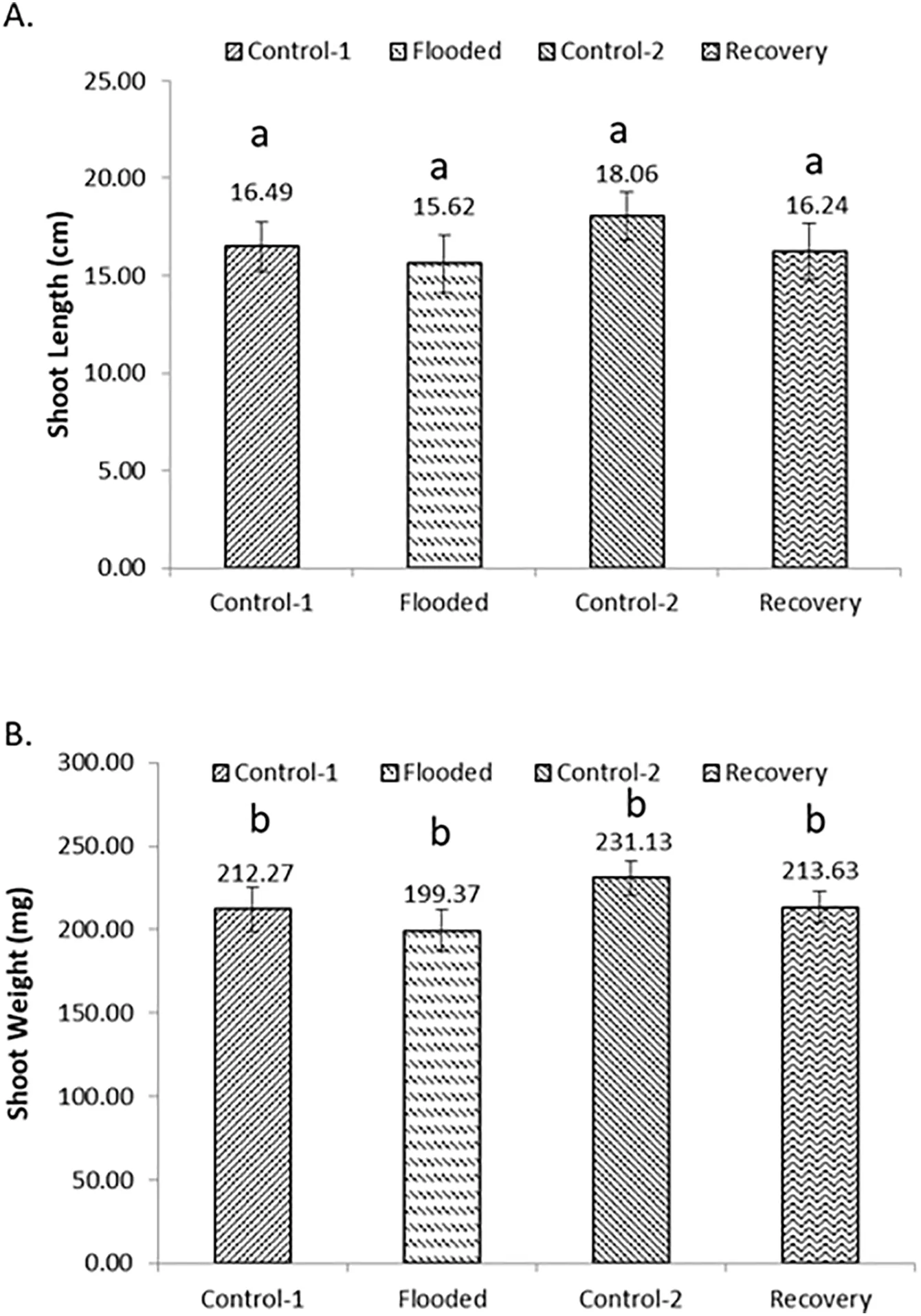
Changes in shoot’s length (A) and weight (B) of wheat in flooded and recovering seedlings. The data is average of 3 replications. Alphabets above the columns indicate statistical changes analyzed through One-Way ANOVA followed by Tukey’s HSD test.

Wheat shoot’s length under flooding stress was measured to be 15.62 cm, while it was 16.49 cm in age-matched control-1 plants. After the recovery phase, the shoot length of the plants that were recovering was 16.24 cm (Fig 3A). Similarly, weight reduction was observed in the shoot. Following 7 days of flooding, a weight reduction of 199.37 mg was observed in flooded shoots, compared to 212.27 mg in control-1 plants. The weight of the shoot that recovered was 213.63 mg, whereas the weight of the shoot in control-2 was 231.13 mg (Fig 3B). The results indicated that flooding stress adversely affects shoot growth.

### 3.3. Morphological changes in root length and weight under flooding stress and recovery durations

**Fig 4:**
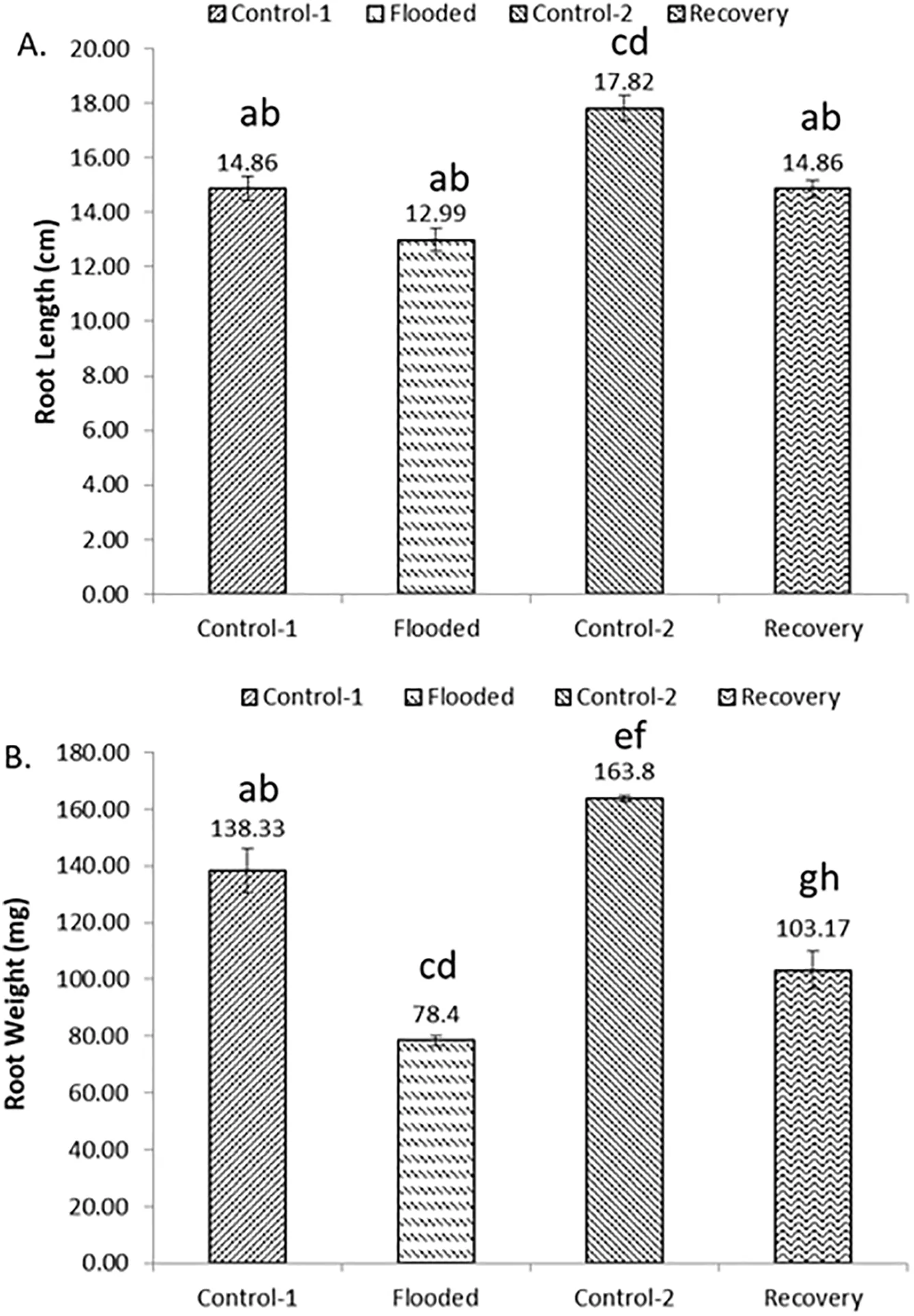
Changes in root’s length (A) and weight (B) of wheat in flooded and recovering seedlings. The data is average of 3 replications. Alphabets above the columns indicate statistical changes analyzed through One-Way ANOVA followed by Tukey’s HSD test.

Length of root subjected to flooding stress was reduced to 12.99 cm in comparison to 14.86 cm in control-1 plants. Following the recovery phase, root length measured was 14.86 cm, compared to 17.82 cm in control-2 plants (Fig 4A). Root length recovery was observed throughout a 7-day recovery period. Flooded roots had a lower weight of 78.4 mg as compared to 138.33 mg in age-matched control-1 plants. The root weight after the recovery period following the flood was 103.17 mg (Fig 4B).

### 3.4. Changes in relative water content (RWC) of wheat under flooding stress and recovery durations

**Fig 5:**
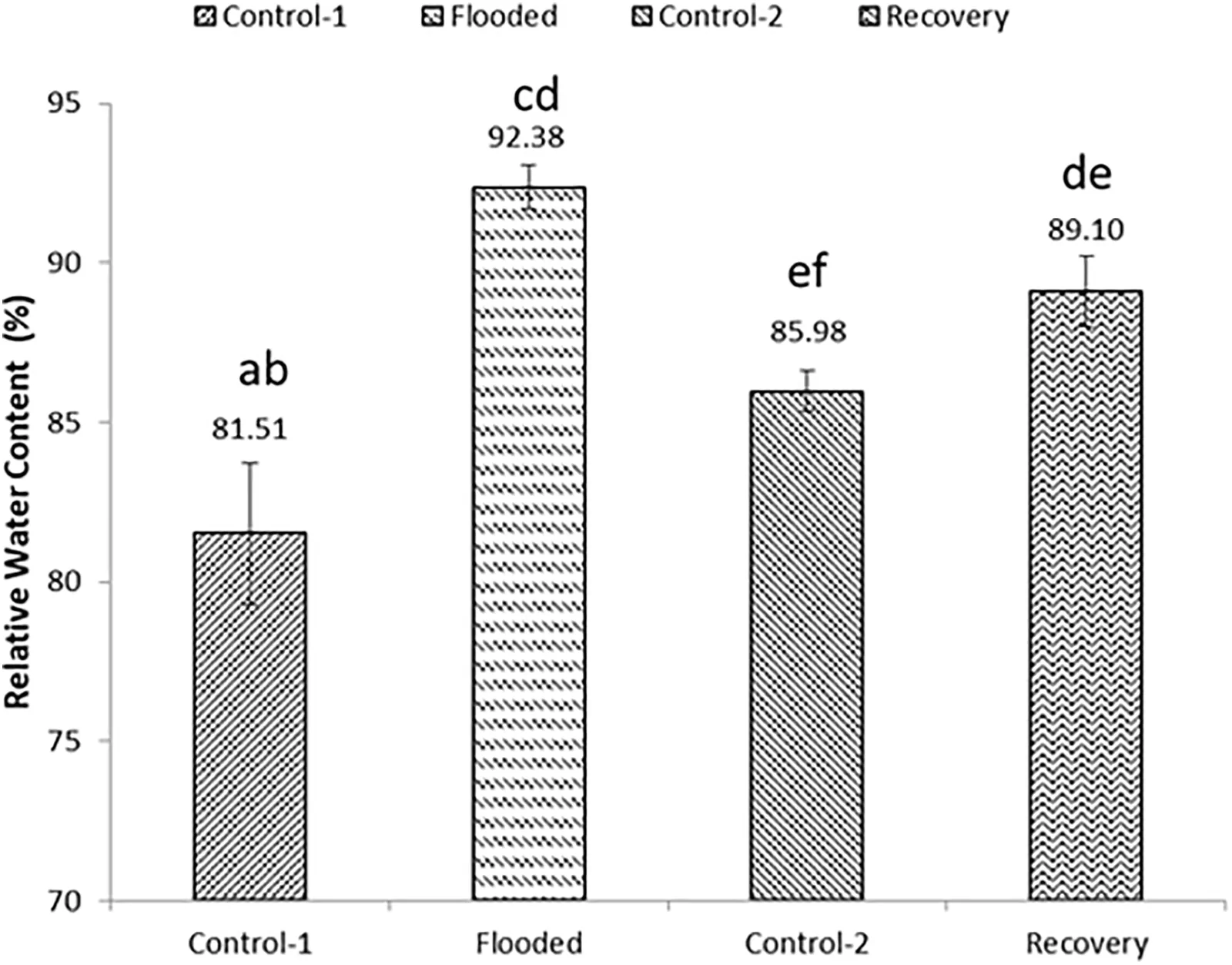
Relative water content changes in control, flooded and during post-flooding recovering seedlings. Alphabets above the columns indicate statistical changes analyzed through One-Way ANOVA followed by Tukey’s HSD test.

RWC in flooded wheat shoots increased to 92.38% as compared to 81.51% in same-aged control-1 seedlings (Fig 5). After 7 days of post-flooding recovery period, RWC was slightly decreased to 89.10%, as against 85.98% in age-matched control-2 plants. The study revealed that the relative water content percent increase in flooded shoots compared to control-1 plants, while the RWC % in flooded shoots tended to return to normal levels throughout the recovery period.

### 3.5. Changes in ascorbate peroxidase activity under flooding stress and during recovery

**Fig 6:**
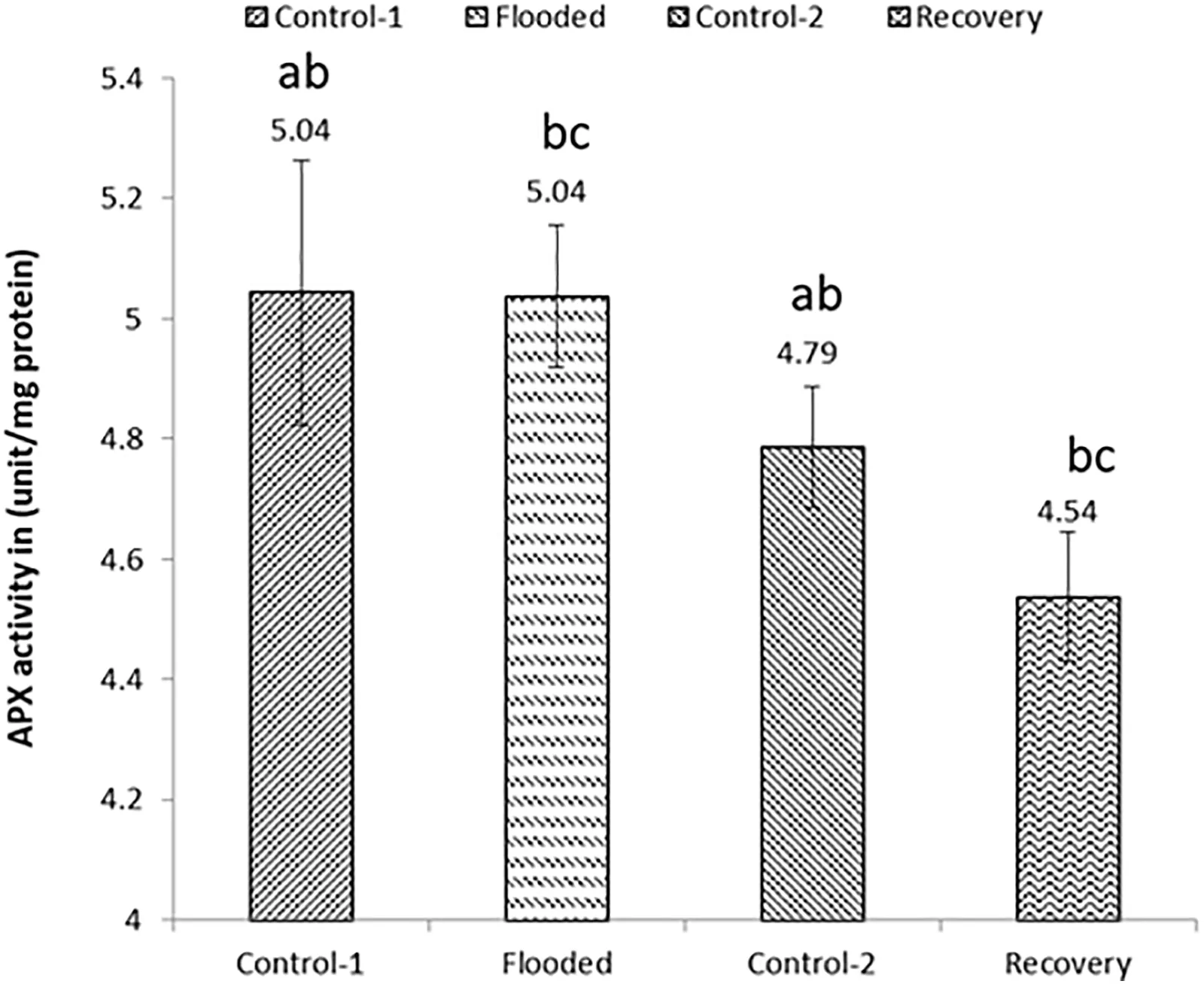
Ascorbate peroxidase activity in flooded and recovering wheat. Data is average of 3 replications. Alphabets above the columns indicate statistical changes analyzed through One-Way ANOVA followed by Tukey’s HSD test.

The APX activity was quantified as 5.04 units/mg protein in control-1 and flooded shoot samples. Following a 7-days recovery phase, the APX activity decreased to 4.54 units/mg protein compared to 4.79 units/mg protein in control-2 plants (Fig 6). The APX activity was found to be similar in both flooded and control-1 plants of the same age. However, it was reduced when measured after 7-days recovery duration.

### 3.6. Fatty acid metabolomics in flooded and recovering wheat

Fatty acids were detected under flooding stress and in the post-flooding recovery period using Gas Chromatography-Mass Spectrometry-based targeted metabolomics. Palmitic acid, oleic acid, linoleic acid, linolenic acid, and oleic acid were detected in all four kinds of samples. Stearic acid was identified in control-1 and recovering plants only. Behenic acid was not detected in control-2 seedlings, as shown in Table 1. The MS raw post run analysis is included in supporting information as S1 File and S2 File. Linolenic acid was identified in the highest amount (51.01% in control-1 and 57.11% in control-2 plants). Palmitic acid was detected as the second major fatty acid in both control-1 (34.54%) and control-2 (39.20%) plants. Linoleic acid was quantified 7.91% and 5.08% in control-1 and control-2 plants, respectively.

**Table 1:** Fatty acids composition (%) in control, flooded and recovering wheat.

Fatty acid (carbons:unsauration)	Retention Time (min.sec)	Control-1	Flooded	Control-2	Recovery	*p* Value
**Palmitic acid** **(16:0)**	25.52	34.54	34.95	39.2	37.76	0.70
**Stearic acid** **(18:0)**	31.43	1.44	ND	ND	5.30	0.64
**Oleic acid** **(18:1)**	31.95	0.95	2.26	2.60	11.51	0.00
**Linoleic acid** **(18:2)**	33.45	7.91	7.20	5.08	7.41	0.01
**Linolenic acid** **(18:3)**	35.80	51.01	56.82	57.11	43.21	0.64
**Behenic acid** **(22:0)**	43.35	1.14	1.25	ND	1.13	0.70

*ND: Not detected.

When compared to control, flooding stress altered the fatty acid composition in seedlings. Palmitic acid, oleic acid, linoleic acid, and linolenic acid were the primary fatty acids identified in flooded conditions. Under flooding stress condition, linolenic acid was found in the highest concentration of 56.82%, whereas oleic acid was detected in the minimum amount of 2.26%. The results revealed 34.95% palmitic acid and 7.20% linoleic acid in flooded plants (Table 1).

Following a 7-day period of recovery after flooding, seedlings were found to possess palmitic acid, stearic acid, oleic acid, linoleic acid, and behenic acid, as shown in Table 1. Palmitic acid, stearic acid, and oleic acid levels increased in post-flooding recovering shoots during the recovery phase compared to both control and flooded shoots. While linolenic acid content (43.21%) tends to decrease during the recovery phase as compared to flooding. Linoleic acid content in post-flooding recovering shoots slightly increased (7.41%) compared to flooded shoots.

### 3.7. Principal component analysis

**Fig 7:**
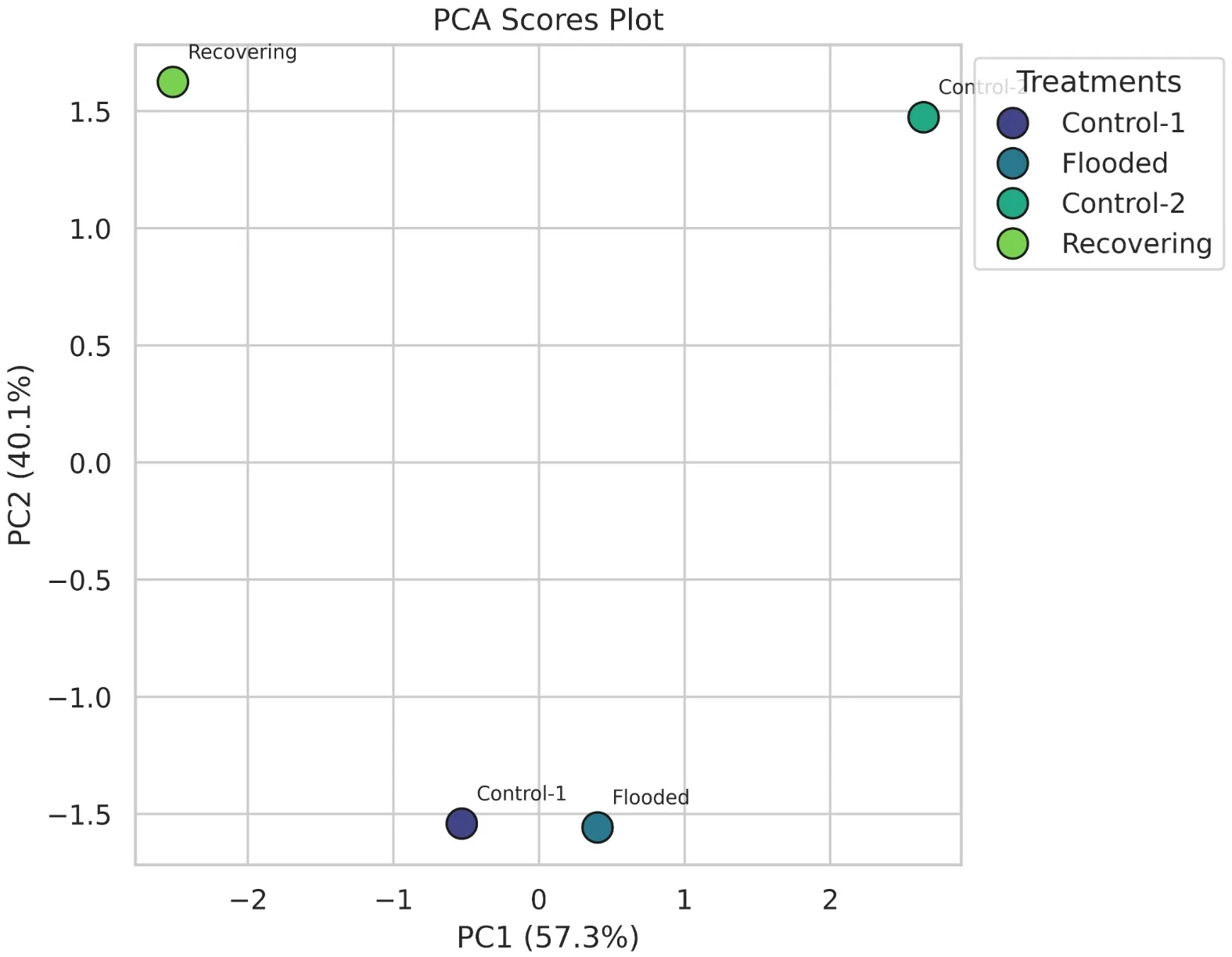
Principal component analysis for control, flooded and recovery time point variables.

The principal component analysis (PCA) of the changes occurring at all analyzed 4 type of samples was performed to reveal their distribution on the plot. PCA1 and PCA2 explains almost 99.9% of variance of the whole data. The plot analyzed the variance of PCA1 and PCA2 where control-1 and flooded are far apart from control-2 and recovery. The control-2 and recovering seedlings are nearly on same scale (Fig 7).

## 4. Discussion

Crops are facing the adverse effects of climate change. These weather variations are blamed for 50% of crop yield losses each year. According to Fathom Global Flooding Map, the flooding events may increase up to 49% by the end of this century [55]. The current study aimed at investigating the impact of flooding stress on the growth, ascorbate activity, and fatty acid composition of the regional wheat variety Atta Habib. The study also investigated the post-flooding recovery potential of wheat after the stress was over. After a 7-days period of recovery following the flooding, wheat potential for recovery was investigated.

The study found that flooding had an impact on the growth parameters of length and weight of roots and shoots of wheat. Flooded plants exhibited reduced length and weight compared to the control group, as seen in Figs 3 and 4. This reduced length and weight of flooded plants was recovered to some extent during the post-flooding recovery phase. Roots and shoots were reported with numerous alterations in biochemical and physiological profiles leading to growth suppression under waterlogging and submergence [20,56,57]. In another study, flooding stress hindered the growth of wheat roots, which partially recovered during the recovery period [16]. Similar growth retardation was recorded in root and hypocotyl of flooded soybean [23,58]. Apricot and plum cultivars showed a decrease in shoot diameter, length, fresh weight, and dry weight when exposed to flooding stress [59]. Flooded plants experienced tissue physical disruption from high water absorption, potentially leading to reduced root and shoot growth [60]. The reported similar studies compliment the findings revealed in our results; suggesting a negative impact on the growth of both roots and shoots, potentially resulting in plant death either if flooding lasts longer or plants not allowed to recover. However, the recovery potential depends on duration of exposure.

The RWC percentage in wheat shoots rose after 7 days of flooding stress as compared to control. Following the recovery time, the RWC in the shoot showed a decreasing trend owing to normalization (Fig 5). A reported study identified a minor reduction in leaf water content and RWC in sorghum plants subjected to waterlogging that was maintained at 3–5 cm above soil (partial submergence) [61]. The waterlogged leaf’s thickness decreased because of alterations in the upper epidermal and mesophyll cells. RWC declined in potato plants under drought stress but increased significantly when the drought-stressed plants were re-watered for 3 days [62]. Increase in foliar RWC was linked to tolerance in salinity-stressed maize [63]. In contrast to drought stress, RWC content in wheat and barley remained high [17]. RWC in wheat was raised in winter wheat facing flooding stress [64]. Waterlogging-tolerant genotypes of onion maintained a higher RWC [65]. In bamboo, Sodium nitroprusside improved resistance to toxic Manganese and chromium by raising RWC in leaf [66]. The partial submergence or only root submergence revealed decrease in levels of RWC owing to inability of plants to carry water upward to shoots due to vascular damage. The rise in shoots RWC in current study may be attributed to complete submergence as plants (including root and shoot) were fully submerged in water in treated group. Moreover, the increased levels of RWC in contrary to those reported under drought stress may be self-explanatory.

The enzyme assay showed that shoot APX activity went down after 7 days of recovery, but there were no changes seen during flooding stress (Fig 6). APX is a key player in scavenging ROS that are induced as a physiological process or due to stresses on plants. It also acts as redox signalling molecule and reduced oxidative damage on plants facing flooding stress [67]. Higher APX activity along with other antioxidant enzymes was reported in flooding-tolerant onion genotypes [65]. APX activity has been linked to flooding tolerance in eggplant and melons [68,69]. Overexpression of eggplant APX raised flooding tolerance in transgenic Arabidopsis [70]. APX activity in soybean roots and shoots declined under prolonged flooding stress after 7–9 days [22]; however, APX activity was increased in mangrove species with raising flood durations [71]. Flooding on rapeseed increased the peroxidase activity by 14.73% in tolerant genotypes and decreased it by 18.03% in susceptible genotypes [72]. In the current study, APX level may have initially been reduced and subsequently increased on the 7th day of flooding stress. APX is a crucial antioxidant enzyme that helps eliminate oxidative compounds produced during oxidative stressors like flooding, salinity, and drought. A recent study on wheat responses to flooding stress found that the activities of peroxidase, superoxide dismutase, and catalase were reduced [16]. The decreased APX activity in recovering plants suggests that once the oxidative stress was scavenged, the enzyme activity returned to normal levels, aiding in plant’s recovery.

**Fig 8:**
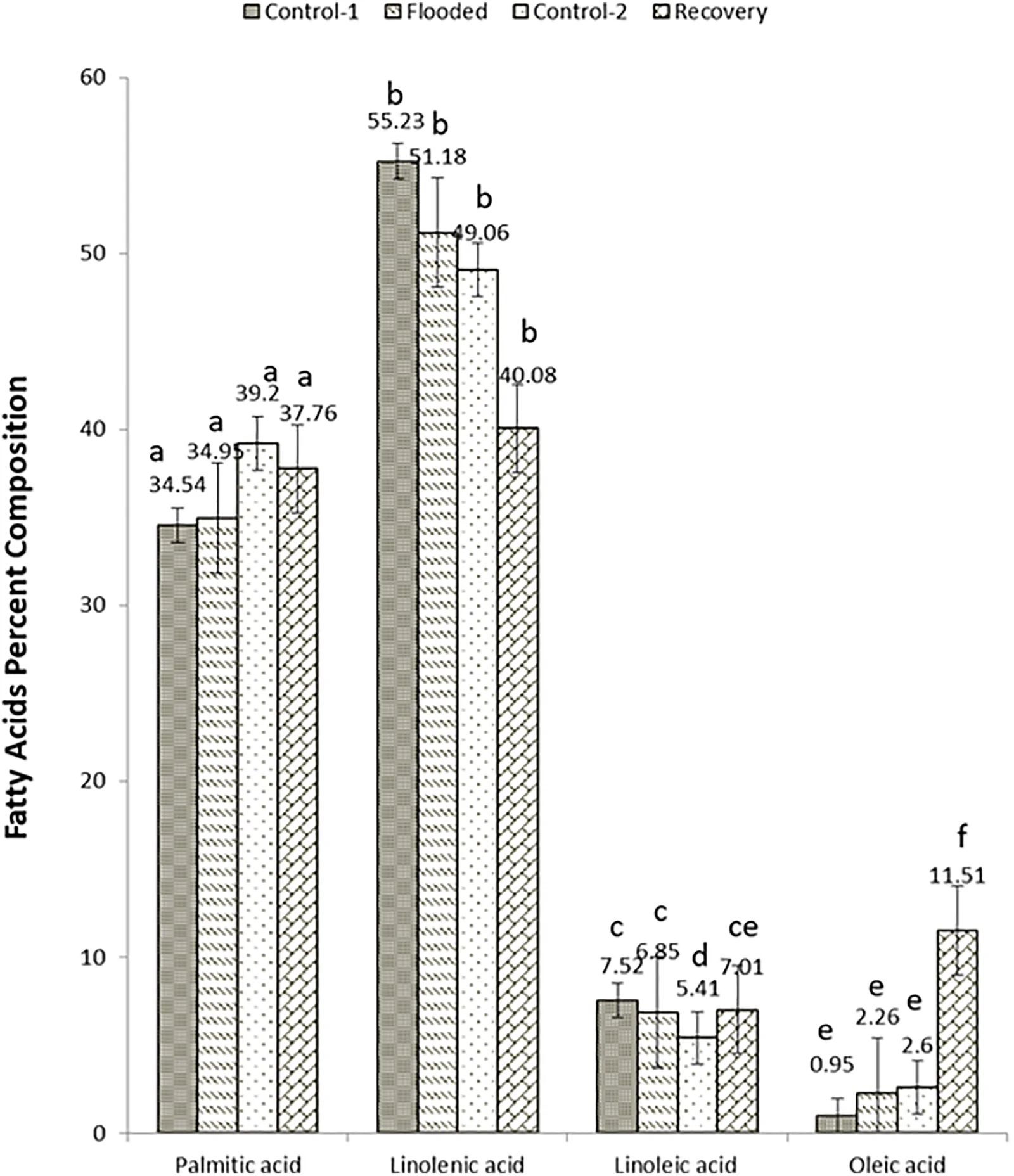
The differences in the percentage composition of key fatty acids identified in control, flooded, and recovering wheat. Alphabets above the column indicate statistical differences as determined by One-Way ANOVA and Tukey’s HSD test. Here changes in a particular fatty acid at a particular stage are compared among themselves to reveal significance or otherwise. Different letters or a combination with no common alphabets indicate statistical significant data at p < 0.05.

In current study, the fatty acid composition showed variable changes after 7 days of flooding stress compared to control plants. Linolenic acid exhibited the highest increase among all fatty acids. Palmitic acid, oleic acid, and behenic acid levels also increased, though to a smaller extent than linolenic acid (Table 1). Palmitic acid concentrations significantly increased after the recovery period in comparison to plants that were flooded. The wheat that was recuperating showed a fivefold rise in oleic acid content. Linoleic acid concentration showed a modest increase relative to both the control and flooded plants. High quantities of linolenic acid were seen during flooding stress but gradually declined during the recovery phase. Palmitic, linolenic, linoleic, and oleic acids were detected in all four kinds of samples (Fig 8).

Flooding induced changes in quality and quantity of metabolites in waterlogged plants [12]. Omics approaches such as metabolomics can offer revealing insights into flooding stress-responsive pathways [73]. Lipid peroxidation is reported due to accumulation of ROS in plants facing flooding stress [74]. The pre-dominant fatty acids found in plant lipids include palmitic acid, stearic acid, oleic acid, linoleic acid, and linolenic acid [75]. C16 and C18 saturated and unsaturated fatty acids produced in the plastids and transported to cytosol, act as precursor for synthesis of very long chain fatty acids that play documented role in plant responses to biotic and abiotic stresses [76]. Previous studies reported that free fatty acids aggravate in plants under stress-induced hypoxia [77,78]. Linoleic acid and linolenic acid were reported to act as precursors for the synthesis of long chain fatty acids, aldehydes, alcohols and esters as well as complex defence molecules that have numerous roles in plant stress responses [44,45]. The concentrations of unsaturated fatty acids such as oleic acid, linoleic acid, linolenic acid significantly increased under cold stress; while the content of palmitic acid and stearic acid decreased [79]. Palmitic acid, linoleic acid content were increased to 11% and 3%, respectively; whereas, stearic acid and oleic acid were decreased to 6% and 11%, respectively in drought-stressed sunflower [80]. Plants alter lipid composition including the balance b/w saturated and polyunsaturated fatty acids in response to stress [78,81]. Higher levels of linolenic acid were linked with drought tolerance and 16 carbon unsaturated fatty acids were associated with post-drought recovery in maize [82].

Lipid breakdown caused disruption of the membrane structure in *Arabidopsis thaliana* subjected to flooding for 3 days [46]. Flooding altered the fatty acid levels and their relative composition in cotton seedlings [83]. Palmitic, linoleic, and linolenic acids were found in elevated concentrations in flooded cotton seedlings. Fatty acid levels rose under oxidative stress, impacting particular mechanisms that help plants withstand challenging environments [84]. In ramie leaf exposed to submergence, lipids formed lipid droplets that were aggregated in cytoplasm, leading to fatty acids accumulation with increasing duration of submergence [49]. A recent study confirmed that submergence/flooding induced hypoxia impairs fatty acid biosynthesis by inactivating long chain acyl-CoA synthetases and accumulation of unsaturated acyl-CoAs [25]. Genes related to linoleic acid and linolenic acid metabolism were differentially regulated in tolerant and susceptible rapeseed genotypes, suggesting their involvement in flooding tolerance mechanism [72]. Lipid saturation and composition remodelling greatly affects plant stress responses through changing fluidity and permeability of plant cell membranes [85]. The results of the current study as well as those reported from other studies reveal a clear cut involvement of fatty acids in abiotic stress responses at different biochemical and signalling levels. All the above discussed reports in compliance with results of current study, document the evident roles of fatty acids in stress responses in general and flooding in particular

## 5. Conclusions

The study results indicate that flooding had a negative impact on plant growth by retarding the lengths and weights of both roots and shoots. These retarded growth parameters tended to recover during the post-flooding recovery period. Relative water content increased under flooding stress but was normalized during the recovery phase. Flooding caused oxidative damage to the plant, against which the antioxidant APX enzyme was activated to reduce the oxidative damage and scavenge the toxic peroxides. Fatty acid abundances were variably changed under flooding stress and recovery. As fatty acids play important protective roles in the membranes of cells as well as acting as signalling molecules, their content changes indicate adjustments to protect cellular membranes, regulate the transport of solutes and solvents across membranes, and convey signals for other protective measurements that may help plants recover in the post-flooding recovery period. In a nutshell, the results revealed that flooding stress suppressed the growth of wheat, that shown a recovery potential in the post-flooding period. In alignment with growth parameters, the activity changes in APX enzyme and variations in fatty acids concentrations revealed necessary adjustments for coping with the flooding stress and signalling for survival. The limited one duration flooding treatment and single wheat cultivar remained the limitation of the current pilot study. Hence, it is further recommended that flooding stress experiments should be extended with more diverse wheat germplasm and with variable flooding durations and recovery periods to reveal extensive insights.

## Supporting information

S1 File GC-MS post run analysis of mass spectra. (DOCX)

S2 File GC-MS post run analysis for final identifications and quantifications. (DOCX)
