# Supplementary material for: Investigation of changes in the profile of fatty acids and growth traits in response to waterlogging/flooding stress
Source: PLoS One. 2026 Aug 28;21(8):e0355293. doi: 10.1371/journal.pone.0355293 (PMC13524277; doi:10.1371/journal.pone.0355293)
Supplement: Supplementary file 2 [file pone.0355293.s002.docx]

**S2 File. GC-MS post run analysis for final identifications and quantifications.**
